# Intraperitoneal infusion of ex vivo-cultured allogeneic NK cells in recurrent ovarian carcinoma patients (a phase I study)

**DOI:** 10.1097/MD.0000000000014290

**Published:** 2019-02-01

**Authors:** Janneke Hoogstad-van Evert, Ruud Bekkers, Nelleke Ottevanger, Nicolaas Schaap, Willemijn Hobo, Joop H. Jansen, Leon Massuger, Harry Dolstra

**Affiliations:** a Department of Obstetrics and Gynecology; b Department of Medical Oncology; c Department of Hematology, Radboud University Medical Center; d Department of Laboratory Medicine, Laboratory of Hematology, Radboud Institute of Molecular Life Sciences, Nijmegen, The Netherlands.

**Keywords:** immunotherapy, intraperitoneal therapy, natural killer cells, recurrent ovarian cancer

## Abstract

**Introduction:**

Recurrent ovarian carcinoma has dismal prognosis, but control of disease and prolonged survival are possible in some patients. The estimated 5-year survival is 46% for all stages of ovarian cancer, and only 28% for metastasized disease. Notably, the majority of women with ovarian cancer are diagnosed with stage III or IV disease with a high recurrence rate. As most women with relapsed or metastatic cancer will die of progressive disease, there is an urgent need for novel therapeutic strategies. The primary aim of our study is to evaluate safety and toxicity of intraperitoneal infusion of ex vivo-expanded natural killer cells (NK), generated from CD34+ umbilical cord blood (UCB) progenitor cells, with and without a preceding non-myeloablative immunosuppressive conditioning regimen in patients suffering from recurrent ovarian cancer. The secondary objectives are to compare the in vivo lifespan, expansion, and biological activity of intraperitoneally infused NK cell products with or without preparative chemotherapy, as well as evaluate effects on disease load.

**Methods:**

In this phase I safety trial, 12 patients who are suffering from recurrent ovarian cancer, detected by a significant rise in serum level of CA-125 on two successive time points, will be included. Prior to UCB-NK cell infusion, a laparoscopy is performed to place a catheter in the peritoneal cavity. The first cohort of three patients will receive a single intraperitoneal infusion of 1.5-3×10^9^ UCB-NK cells, generated ex vivo from CD34+ hematopoietic progenitor cells obtained from an allogeneic UCB unit, without a preparative chemotherapy regimen. The second group of three patients will be treated with a similar dose of UCB-NK cells following a preparative four days non-myeloablative immunosuppressive conditioning regimen with cyclophosphamide and fludarabine (Cy/Flu). If no severe toxicity is seen in these 6 patients, an extension cohort of 6 patients will be included to answer the secondary objectives.

**Discussion:**

This study investigates the safety of a promising new cellular therapy in a group of patients with a poor prognosis. Demonstration of safety and in vivo expansion capacity of allogeneic UCB-NK cells in the absence of Cy/Flu pretreatment will provide rationale for UCB-NK cell infusion after regular second-line chemotherapy.

## Abstract 2

Supplemental Digital Content is available in the text

## 1 Introduction

Recurrent ovarian carcinoma (OC) remains largely incurable, but control of disease and prolonged survival can be achieved in some patients. The estimated 5-year survival is 46% for all stages of ovarian cancer, and only 28% for metastasized disease.^[1]^ Notably, nearly 70% of women with OC present with stage III or IV disease, for which the recurrence rate is 60%–70%. As most women with relapsed or metastatic cancer will die of progressive disease, there is an urgent need for novel therapeutic strategies. Current therapy of relapsed disease consists of 6 cycles of palliative chemotherapy, which are not always completed due to side effects or early progression. New drugs include the antibody bevacizumab and the tyrosine kinase inhibitor cediranib targeting the VEGF axis, which are used in combination with gemcitabine and carboplatin chemotherapy. Although these combinations show improvement of progression-free survival (PFS), they do not prolong overall survival.^[2,3]^ Interestingly, accumulating evidence indicates that presence of immune responses has prognostic value in OC. Infiltrating CD8+ T cells and CD56+ NK cells can be detected in OC biopsies, and strong infiltration of CD8+ T cells is correlated with prolonged survival.^[4–7]^ As most tumors with infiltrating CD56+ NK cells also contain CD8+ T cells, the precise contribution of these individual populations in mediating antitumor efficacy remains unclear. Nevertheless, enhanced NK cell frequencies in the lymphocyte fraction in ascites of advanced OC patients have been associated with prolonged survival.^[8]^ This emphasizes that OC patients could benefit from immunotherapeutic strategies. Therefore, we exploit a unique immunotherapy consisting of highly powerful allogeneic NK cells ex vivo generated from hematopoietic progenitor cells (HPCs) obtained from umbilical cord blood (UCB).

NK cells are key immune effector cells, capable of effectively killing tumor cells through several killing mechanisms.^[9]^ These include release of cytotoxic granules containing perforin and granzymes, TRAIL-dependent cytotoxicity, and activation of Fas-mediated apoptosis. NK cell activation is tightly regulated by activating and inhibitory signals from cell surface receptors.^[10]^ The inhibitory signals are mediated by HLA class I-binding receptors, including killer cell immunoglobulin-like receptors (KIRs) and CD94/NKG2A. Since HLA class I molecules are often lowly expressed on tumor cells,^[11–13]^ these inhibitory receptors are not engaged. So, in the presence of activating signals, target cells will be killed (i.e., the “missing-self” concept).^[14]^ These activating signals are mediated by a wide array of receptors, including NKG2D, DNAM-1, natural cytotoxicity receptors (i.e., NKp30, NKp44, NKp46), CD94/NKG2C, and KIR with activating intracellular domains. Ligands for these activating receptors are expressed predominantly by “stressed” cancer cells, and not by normal cells. Differential expression of these ligands between tumor and normal cells determines the tumor reactivity of NK cells.

In the evolving era of immunotherapy, NK cells are promising candidates for cellular therapy. Numerous trials have been launched in the past years, exploring infusion of allogeneic NK cell products against various cancers.^[15,16]^ Approximately 70% of these trials target hematological malignancies, particularly acute myeloid leukemia (AML). Adoptive NK cell therapy plus low-dose IL-2 after lymphodepleting cyclophosphamide (Cy)/fludarabine (Flu) conditioning has shown to induce 20%–50% complete remissions in relapsed/refractory AML patients.^[17–20]^ Clinical efficacy correlates with in vivo NK cell persistence and expansion. In addition, NK cell therapy is an emerging therapy for solid tumors, including OC.^[21–23]^ The first trial showed that intravenous infusion of allogeneic NK cells plus low-dose IL-2 after lymphodepleting chemotherapy resulted in transient in vivo expansion of NK cells in recurrent OC patients. Notably, most studies are performed with NK cells enriched from peripheral blood (PB) of haplo-identical donors, which typically contains about 30%–70% NK cells with low activation status.^[17,18,24]^ To generate a more homogenous and well-defined NK cell product, we have developed methodology for the expansion of NK cells from UCB-HPCs.^[25–27]^ Recently, we reported that by applying the aryl hydrocarbon receptor (AHR) antagonist StemRegenin-1 (SR1) and the combination of IL-15 and IL-12, we can generate highly active NK cells with potent reactivity against hematological tumor cells *in vitro* as well as anti-leukemic effects in vivo following intravenous administration.^[27–29]^ Preclinical testing showed that this “next generation” UCB-NK cell product also effectively kills OC cells and spheroids.^[30]^ In previous homing studies in NOD/SCID/IL2Rg^null^ (NSG) mice and patients, it has been observed that a major part of the NK cell product accumulates in the liver and lungs 48 hours after IV infusion.^[31,32]^ Since in OC patients the disease is confined to the peritoneal cavity, IP infusion of UCB-NK cells was explored in NSG mice engrafted with SKOV-3 ovarian tumor nodules in the abdomen. Interestingly, significantly decreased tumor progression and improved survival of OC-bearing mice were observed.^[30]^ These findings illustrate that intraperitoneal UCB-NK cell therapy could be a promising strategy to control OC.

The primary aim of our study is to evaluate safety and toxicity of intraperitoneal infusion of ex vivo-expanded NK cells, generated from CD34+ UCB progenitor cells, with and without a preceding non-myeloablative immunosuppressive conditioning regimen in patients suffering from recurrent OC. Secondary objectives are to compare the in vivo expansion, lifespan, and biological activity of intraperitoneally infused NK cell products in patients treated with or without preparative chemotherapy, as well as evaluate effects on disease load.

## 2 Methods/design

### 2.1 Study objectives

The study is designed as a phase I toxicity study in a series of 12 patients suffering from their second recurrence of ovarian, fallopian tube, or primary peritoneal cancer, detected by an elevated serum level of CA-125 on two successive time points with 28 days in between, reaching a level of more than 35 U/ml, to evaluate:

- safety and toxicity of intraperitoneal CD34+ UCB progenitor-derived allogeneic NK cell infusions with a fixed dose of 1.5–3×10^9^ ex vivo-expanded UCB-NK cells in patients treated with or without preceding immunosuppressive conditioning therapy.

Secondary Objectives:

- evaluation of the in vivo expansion and lifespan of UCB-NK cells following intraperitoneal infusion in patients treated with or without preceding immunosuppressive conditioning therapy.
- exploration of the biological and clinical activity of UCB-NK cell infusion in study participants.

### 2.2 Study design

**Figure 1:**
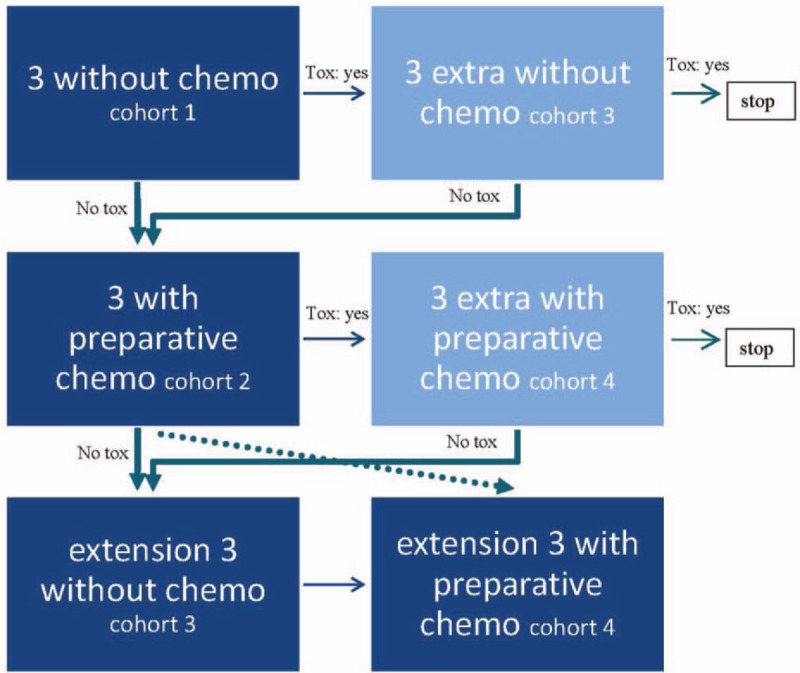
Flow chart study design. In this flowchart, all possible decisions in (dis)continuation of the study are outlined. Toxicity (tox) limits are described in the text.

This is a prospective phase I toxicity study in a single center. In this trial, a total of 12 patients divided over 4 cohorts, suffering from a second recurrence of ovarian, fallopian tube, and primary peritoneal cancer, will be infused with ex vivo-expanded allogeneic UCB-NK cells. In a classical three-by-three design, in the first two cohorts of three patients, the safety and toxicity of intraperitoneally infused UCB-NK cells in the absence or presence of Cy/Flu pre-treatment will be studied. The question whether the chemotherapeutic preconditioning regimen is necessary will be addressed in the extension cohorts of three patients without and three patients with a preparative non-myeloablative immunosuppressive chemotherapy regimen. This means that cohort 1 gets NK cell without Cy/Flu pretreatment, cohort 2 NK cells with Cy/Flu, cohort 3 NK cells without Cy/flu, and cohort 4 again NK cells with Cyflu pretreatment, see Fig. 1.

### 2.3 Study schedule

The trial starts in the beginning of 2019. The estimated time of accrual is 30 months. The estimated study completion date is July 2021, for final data collection and secondary outcome measures.

### 2.4 Statistics

This study has a three-by-three design, with 2 extension cohorts of 3 patients. A total of 3 patients will be included into each treatment arm in order to appropriately determine UCB-NK cell treatment-related toxicity. To answer the other key question whether Cy/Flu preconditioning is required for successful UCB-NK cell engraftment and expansion in the peritoneal cavity, we will perform statistical analyses on the immunomonitoring results. The proportion of patients with NK cell expansion as well as the number of NK cells in the peritoneum and blood will be statistically compared between cohorts 1 and 3 (without Cy/Flu) and cohorts 2 and 4 (with Cy/Flu) using the Mann–Whitney U-test. Using the statistical program R, we calculated that a group size of 6 patients per cohort is sufficient to evaluate whether preconditioning is required or not. We estimated that preconditioning results in a frequency of 50 ± 20% UCB-NK cells IP at day 7 and/or 14 determined by flow cytometry and DNA chimerism analyses. In case NK cell expansion is >3-fold lower, i.e., 15%±20%∗ in patients not treated with preconditioning, this will be a statistically less effective treatment at a significance level of 0.05 and a power of 0.80. So after the classic three-by-three toxicity study, we will continue with the extension cohort of three patients per group. If no statistical difference is observed in the total of 6 patients treated with versus 6 patients treated without preconditioning, we will conclude that preconditioning is not required for IP UCB-NK cell engraftment and expansion in OC patients. In case <5% of the peritoneal CD56+CD3-cell fraction is of UCB-NK origin in preconditioned patients (cohort 2), this is considered unsuccessful and the trial will be stopped. In that case, the extension cohort will not be exposed to an ineffective experimental treatment and chemotherapy. The secondary goal of clinical (CA-125) response has a descriptive nature. Data management will be conducted in Castor (Castor EDC, CIWIT B.V. Amsterdam) in collaboration with our clinical trial office.

### 2.5 Recruitment

The trial is expected complete recruitment within 30 months. Participants will be recruited from the Radboud University Medical Center and referred from the oncologic region, the south east Netherlands. After obtaining informed consent, patients will be screened for inclusion and exclusion criteria (Table 1). After fulfilling all eligibility criteria, patients will be enrolled in the study.

**Table 1:**
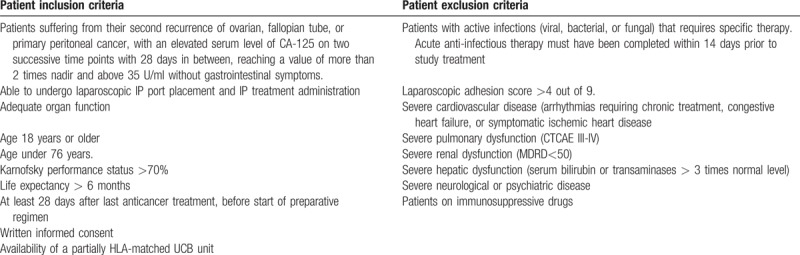
In- and exclusion criteria.

### 2.6 Study intervention

This study is a phase I toxicity trial, using ex vivo-generated NK cells from CD34+ UCB cells of allogeneic partially HLA-matched donors. These ex vivo-generated allogeneic UCB-NK cells will be infused intraperitoneally into patients suffering from recurrent ovarian, fallopian tube, or peritoneal cancer treated with or without Cy/Flu preconditioning. This immunosuppressive conditioning regimen is often applied with intravenous NK cell infusion to prevent direct rejection, but might not be required in case of IP administration. The investigational CD56+CD3- UCB-NK cell products will be ≥70% pure and almost devoid of CD3+ T cells (i.e., <1×10^5^ cells/kg body weight), thereby minimizing the risk of allogeneic T cell-mediated GVHD. NK cell therapy is combined with IL-2 cytokine support, at a dose of 6×10^6^ units/dose, 3 times a week with a total of 6 dosages. Study participants will undergo clinical and immunological evaluation

#### 2.6.1 Laparoscopy

To allow infusion of the UCB-NK cells in the abdominal cavity, a single lumen 9.6fr attachable silicone catheter (Bard Medical) is placed per laparoscopy. If there are too many adhesions to reach 5 out of 9 compartments of the abdominal cavity, no catheter is placed and this patient will be excluded from further study treatments.

#### 2.6.2 Pretreatment

All patients will receive an intraperitoneal infusion of 1.5–3×10^9^ allogeneic NK cells generated ex vivo from CD34+ cells obtained from a UCB unit. This dosage is based on results of published trials with PB-enriched NK cells and our previous UCB-NK trial in elderly AML patients.^[29]^ Miller et al are currently executing a trial on peritoneal infusion of enriched PB-NK cells with IL-2 support in ovarian carcinoma patients (NCT02118285) and a trial on intraperitoneal FATE-NK100 cells (NCT03213964). IP infusion might be a valuable strategy, since the immunologic environment in the abdomen is completely different from the situation in blood. The hypothesis is that our UCB-NK cell product can survive and expand in the abdominal cavity without requiring a preparative lymphodepleting chemotherapy regimen. However, lymphodepleting chemotherapy is currently the standard clinical practice for adoptive cellular therapy in cancer patients. In the first cohort, toxicity of UCB-NK cell therapy with IL-2 support will be monitored. The second cohort of 3 patients will receive a non-myeloablative immunosuppressive preparative regimen of cyclophosphamide (900 mg/m^2^/day) and fludarabine (30 mg/m^2^/day) on days 6, 5, 4, 3. Ample experience with this preparative conditioning regimen is present in our transplant center where patients suffering from multiple myeloma and high-grade Non-Hodgkin's lymphoma are treated with this regimen.^[29,33]^ In addition, in our trial with the first generation UCB-NK cell product in elderly AML patients, no severe toxicity was observed. Currently, Cy/Flu is the standard preparation for cellular adoptive therapy. To evaluate potential differences in expansion and lifespan of UCB-NK cells in the abdominal cavity in patients treated with or without myeloablative preconditioning, an extension cohort of 3 patients per group will be performed, thereby obtaining 6 patients per group.

#### 2.6.3 UCB-NK cells

In eligible patients, UCB-NK cell products will be administered IP at a dose of minimum 1.5×10^9^ and maximum 3×10^9^ UCB-NK cells. In the first cohort, UCB-NK cells will be infused three days after the Cy/Flu regimen. The UCB-NK cell dosage is based on our study in elderly AML, where we demonstrated that Cy/Flu conditioning followed by administration of the UCB-NK cell product at a dose ≤3×10^7^/kg body weight, i.e., ≤3×10^9^ total cells in a patient of 100 kg, was safe and did cause neither GVHD nor severe toxicity. Here, we want to investigate whether UCB-NK cells are safe to administer intraperitoneally in OC patients, as this is the standard route of chemotherapy administration in ovarian carcinoma therapy to allow better targeting of the intraperitoneal disease. Intraperitoneal NK cell infusion may favor NK cells to quickly interact with the tumor cells, without being trapped in the liver or lungs. Since the immunologic environment in the abdominal cavity is different from the environment in blood and lymphoid organs, the hypothesis is that the UCB-NK cells may survive and expand in the abdominal cavity without the preparative regimen. Based on our previous clinical trial and the route of administration, we do not expect severe toxicity of our allogeneic NK cell product.

#### 2.6.4 IL-2

IL-2 (proleukin) will be co-administered IP to support NK cell expansion and survival.

### 2.7 Assessment

Patients will be monitored closely for side effects. This includes physical examination, blood tests, and peritoneal fluid collection. All patients will be evaluated intensively for toxicity caused by the conditioning regimen according to the CTCAE toxicity criteria and Glucksberg GVHD grade.^[34]^

### 2.8 Translational research

To study the immunological response, we will determine the percentage and absolute number of allogeneic UCB-NK cells in peripheral blood (PB) and peritoneal fluid (PF) after infusion. These analyses will be done by flow cytometry and DNA chimerism analyses. In parallel, we will determine the donor NK cell engraftment and IL-15 plasma level in blood in the context of NK cell survival and expansion in the peritoneal cavity. Finally, we will assess cytolytic activity of NK cells, isolated from peritoneal fluid, using functional assays.

To assess these biological parameters, PB samples will be taken on day 0 (prestudy sample) and day 1, 3, 7, 14, 21, 28 and 56 after IP-NK cell infusion. PF samples will be obtained at day 0, 7, 14, and 28. The following immunological tests will be conducted on blood and IP-wash samples:

#### 2.8.1 Phenotypic analyses of NK cells

To study the effect of treatment on leukocyte composition, immunophenotyping will be directly performed on PB samples and PF samples collected following UCB-NK administration. Using a whole blood phenotype assay, we will quantify the proportion of the following lymphocyte subsets: NK cell subsets (CD3-CD56^bright^ and CD3-CD56^dim^), NKT cells (CD56+CD3+), T cell subsets (CD3+CD4+ and CD3+CD8+ T cells in combination with CCR7, CD45RA, CD27, CD25, CD127, FoxP3), and B cells (CD19+). Absolute numbers of circulating lymphocyte subsets will be determined by single-platform flow cytometry analyses using counting beads. Effective NK cell expansion is defined by presence of ≥5% donor-derived NK cells within the circulating and/or peritoneal cell fraction at day 7 after infusion. In addition, multicolor flow cytometry panels will be used to examine the frequency and phenotype leukocyte subsets in the IP-wash samples. Next to the above mentioned lymphocyte subsets, we will determine the proportion of macrophages and CD45-tumor cells. Furthermore, in both blood and IP-wash samples, the CD56+CD3-NK cell fraction will be analyzed for the expression of KIRs (CD158a, CD158b, CD158e), c-type lectin receptors (CD94/NKG2A, NKG2C, NKG2D), NCR (NKp30, NKp44, NKp46), activation markers (DNAM-1, CD69, CD16), cytotoxic mediators (Perforin, TRAIL), cytokine receptors (CD25, CD127, CD122), and homing receptors (CXCR3, CD62L).

#### 2.8.2 Enumeration of donor-derived NK cells

To assess in vivo expansion and persistence of the infused NK cells, we will perform standard chimerism analysis on DNA derived from PB and IP-wash samples. DNA will be extracted from blood and IP-wash samples (Qiagen DNA blood mini kit) and used to determine the proportion recipient versus donor cells by real-time quantitative PCR-based single nucleotide polymorphisms (SNP) that are discriminative for the UCB donor and OC patient. Patient and donor DNA, collected before infusion, will be used to identify differential SNP in a panel of polymorphic genes. Effective NK cell expansion is defined by presence of ≥5% donor-derived NK cells within the circulating and/or peritoneal cell fraction at day 7after infusion.

#### 2.8.3 Detection of cytokines in plasma

Pre- and postinfusion plasma and peritoneal wash samples will be evaluated for cytokine levels by a commercial ELISA or LUMINEX assay. In cohorts 2 and 4, plasma and PF collected before and after Cy/Flu conditioning will be examined for enhanced levels of endogenous cytokines (IL-15, IL-7). In addition, we will determine the levels of inflammatory cytokines (IFN-γ, TNF-α, IL-6). Cytokine levels will be correlated with absolute lymphocyte and NK cell counts, as well as NK cell chimerism.

#### 2.8.4 Functional activity of UCB-NK cells

To enumerate the number of NK cells reactive against the patient OC cells, we will analyze CD107a degranulation and intracellular IFN-γ using flow cytometry. For this, PBMC and peritoneal wash samples will be cocultured with different OC cell lines (e.g., SKOV-3, OVCAR, IGROV1), patient-derived ovarian carcinoma cells, and/or K562 cells (positive control). After overnight incubation, the percentage CD107a+ and/or IFN-γ+ cells within the CD56+CD3-NK cell population will be determined by flow cytometry. In parallel, we will evaluate the amount of IFN-γ produced by the stimulated NK cells using ELISA.

#### 2.8.5 Phenotypic analysis and enumeration of tumor cells present in peritoneal fluid

To study the effect of UCB-NK cell infusion on the frequency and phenotype (activating and inhibitory ligands) of tumor cells in the peritoneal fluid, immune phenotyping will be performed on the PF samples obtained before and after UCB-NK administration. This analysis will be performed on PF samples directly after collection.

#### 2.8.6 Response on residual disease in treated patients

The effect of NK cell infusion on measurable residual disease will be investigated by determining CA-125 serum levels.

## 3 Discussion

This study investigates the feasibility and safety of a promising new cellular therapy in a group of OC patients with a poor prognosis. Previous studies exploring different allogeneic NK cell products showed mild toxicity profiles in various cancers. We and others demonstrated promising clinical effects in AML patients treated with allogeneic NK cell adoptive transfer. However, for OC, this has not been clearly demonstrated yet. Here, we present the first study investigating our allogeneic UCB-NK cell product, which is highly activated and exhibits profound cytotoxic potential, in OC patients following intraperitoneal infusion. This provides us with the unique opportunity to monitor immunological responses in the abdominal cavity by consecutive sampling via an IP catheter.

To explore the therapeutic potential of this UCB-NK cell product in OC patients, and to conclude whether a myeloablative immunosuppressive chemotherapeutic regimen is necessary in IP cellular therapy, there is the need to conduct this phase 1 trial. The results of this study will provide important information on the benefit and potential pitfalls of IP cellular therapy, and give insight in the immunological OC environment during immunotherapy through the repeated peritoneal fluid sampling. Demonstration of safety of UCB-NK cell therapy and NK cell expansion in the absence of Cy/Flu pretreatment could provide rationale for UCB-NK cell infusion after regular second-line chemotherapy.

## Acknowledgments

The authors would like to thank the Dutch Cancer Society for funding. We would like to thank the Radboudumc Technology Center Clinical Studies for all assistance in the logistics of the trial.

## Author contributions

JH and HD wrote the manuscript. NO, RB, MS, WH, JJ and LM revised the manuscript and advised in trial setup.

**Funding acquisition:** Harry Dolstra, Janneke Hoogstad-van Evert and Leon Massuger.

**Investigation:** Janneke Hoogstad-van Evert.

**Project administration:** Janneke Hoogstad-van Evert.

**Supervision:** Nelleke Ottevanger, Leon Massuger, Harry Dolstra.

**Writing – original draft:** Janneke Hoogstad-van Evert, Ruud Bekkers, Harry Dolstra.

**Writing – review & editing:** Ruud Bekkers, Nelleke Ottevanger, Nicolaas Schaap, Willemijn Hobo, Joop H. Jansen, Leon Massuger, Harry Dolstra.

Janneke Hoogstad-van Evert orcid: 0000-0001-9838-0977.

## Supplementary Material

Supplemental Digital Content
