# Supplementary material for: Intraperitoneal infusion of ex vivo-cultured allogeneic NK cells in recurrent ovarian carcinoma patients (a phase I study)
Source: Medicine (Baltimore). 2019 Feb 1;98(5):e14290. doi: 10.1097/MD.0000000000014290 (PMC6380776; doi:10.1097/MD.0000000000014290)
Supplement: Supplemental Digital Content [file medi-98-e14290-s001.doc]

**Structured Risk Analysis**

1. Level of knowledge about mechanism of action

The mechanism of action of NK cells is that NK cell can kill cancer cells without prior sensitization, and that NK cells don’t attack healthy cells. Toxicity that might be expected from UCB-NK cell infusion includes abdominal pain, tumor lysis syndrome and GVHD (see appendix 3 and ). The preparative chemotherapy regimen can induce fever, chills, myalgias, malaise and allergic reactions and additionally pancytopenia requiring transfusions, increasing the probability of (severe) infections. Toxicities associated with IV IL-2 are as follows: flu-like syndrome (fever, chills, tiredness, headache, muscle and joint pain), low blood pressure, nausea, diarrhea, weakness, fluid retention and weight gain, hypothyroidism, abnormal liver function test.

1. Previous exposure of human beings with the test product(s) and/or products with a similar biological mechanism

The UCB-NK cell dosage is based on our PLMA25 study in which we show that following Cy/Flu conditioning UCB-NK cell products could be administered to AML patients up to 3x10^7^/kg body weight i.e. 3x10^9^  cells in total for a patient of 100kg, without induction of GVHD or severe toxicity. Furthermore, we want to investigate whether UCB-NK cells are safe to administer intraperitoneally. Chemotherapy is administered IP in standard ovarian carcinoma therapy, for better targeting of the intraperitoneal disease. Miller et al are currently executing an intraperitoneal NK cell trial and has not observed any toxicity of the PB-NK cells up to a dose of 3x10^9^ cells with preparative Cy/Flu chemotherapy.

In a study in women with platinum resistant or refractory ovarian cancer the IP administration of single agent IL-2 weekly at dose higher than used in this study was generally well tolerated. Grade 1 and 2 constitutional symptoms (flu-like symptoms, GI and neurological) were most common, but controlled with medication and not requiring dose reduction.

1. Can the primary or secondary mechanism be induced in animals and/or in *ex-vivo* human cell material?

We have demonstrated that an improved second generation UCB-NK cell product can efficiently attack OC spheroids as well as OC tumors in a preclinical mouse model after intraperitoneal (IP) infusion. To be able to see the effect of UCB-NK cells on ovarian carcinoma in a human immune system in the abdominal cavity, it is necessary to perform this study. We did our best to perform all pre-clinical studies possible.

1. Predictability of effect

Because of our PLMA study and the study of Miller in a comparable patient category, we have confidence that we won’t see unexpected toxicity, and we hope to see some effect. All safety measures and monitoring is available for unexpected toxicity.

1. Can effects be managed?

During conditioning and after infusion, patients will be observed under supervision of experienced hematologists, oncologists and gynecologists during hospitalization. All patients will be seen under supervision of an experienced gynecologists or hematologist in the outpatient clinic twice weekly until recovery of the bone marrow. So appropriate measures can be taken in case of any unforeseen reaction to the cells takes place.

A safety monitoring board will be installed, composed of 2 independent physicians with knowledge of the field of research. For this purpose a standardized monitor form will be used (see appendix). This will be Hans Nijman, gynaecologic oncologist, Groningen UMC, and Annemarie Thijs, Medical oncologist Catharina Hospital Eindhoven. Both are independent from the study team and from the sponsor.

Monitoring frequency is planned as follows:

- an initiation visit

- First visit 7 days after NK cell infusion of first and third patient of cohort 1 and 2.

- Second visit 28 days after NK cell infusion of first and third patient of cohort 1 and 2.

- Third visit after day 28 of patient (nr 9)

- a close out visit after completion of last patient.

At initiation visit monitoring includes the following:

- completeness of the Study Master File

- check procedures: availability of SOP

See attachment for details.

# APPENDIX

**STUDY MONITOR FORM** (completed at study initiation visit)

|  | **Comments** | **Monitor Paragraph** |
| --- | --- | --- |
| Is the signed protocol, including IB and CMO approval available at study centre |  |  |
| Does the study master file contain all essential documents including a list of all members of the study staff, with their signatures |  |  |
| Are all CV’s of study staff members available at study centre |  |  |
| Are CRFs available at study centre |  |  |
| Are normal values for lab tests available at study centre |  |  |
| Has the project been properly introduced at CRCN, department of clinical chemistry and clinical pharmacy? |  |  |

Subject number:

| Standard checks | **Comments** | **Monitor Paragraph** |
| --- | --- | --- |
| Volunteer complies with inclusion/exclusion criteria |  |  |
| Source document contains copy of informed consent, all lab results, and completed evaluation form |  |  |
| CRF is filled in completely |  |  |
| Monitoring of all grade 3-4 AE and all SAE |  |  |
| Primary endpoint |  |  |

| **Datamanagement Plan**  Applicable for investigator initiated studies covered by the Medical Research Involving Human Subjects act | | | |
| --- | --- | --- | --- |
| **NL-number** | | NL60937.000.17 | |
| **Title** | | Intraperitoneal infusion of ex-vivo cultured allogeneic natural killer cells in recurrent ovarian carcinoma patients, a phase 1 study | |
| **Short title** | | INTRO | |
| **DMP version number and date** | | Version 3 | Date: 17-10-18 |
| **Coordinating investigator / project leader** | | Dolstra, Harry  *(last name, first name)* | |
| **Data Coordinating Center / Data Management Contact(s)** | | Trialbureau medical oncology: Peter van Essen  *(last name, first name)* | |
| **Type of study** | | Single centre  Multi centre, number of sites:  Total number of patients (approximately): 12 | |
|  |  | Non randomized  Randomized controlled  If randomized controlled:  Open label  Single blind Double blind | |
| Please indicate that the following applies:  We acknowledge that the handling of the study data is the responsibility of the coordinating investigator/project leader  This study and all persons involved have knowledge of and comply to the most recent version of:  the Dutch Personal Data Protection Act (De Wet Bescherming Persoonsgegevens, Wbp).  the Code of proper Coduct (Code Goed Gedrag – FEDERA)  Instructions for and more information about the questions below can be found on the CRCN Data Management intranet pages. | | | |
| **Name Data Management system used** | Castor EDC | | |
| **Is this a validated system** | [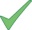](https://www.google.nl/url?q=http://vector.me/search/check-mark&sa=U&ei=33AgU478B6Wd0wXav4GIDA&ved=0CC0Q9QEwBQ&usg=AFQjCNG2vSjGtoxg-z3Ic-vu90eu4Ni7Wg) Yes | | |
| **Describe the types of data, the sources and flow of collection and storing of data (number of steps dependent on study)** | | Step | Type  *e.g.: patient data, life style items, genetic data, lab results,....* | Source  *e.g.: EPIC, paper, lab system, measuring device,.....* | System/format  *e.g.: MACRO, Castor, Excel, paper, SPSS, .....* | | --- | --- | --- | --- | | 1 | Patient data | Epic | Castor | | 2 | Patient Reported Outcome | Epic/paper | Castor | | 3 | lab results | Epic | Castor | | 4 | immunology results | measuring device/digital | Castor | | 5 | radiologie | epic | Castor | | | |
| **Location of and access to the study data**  **1. paper**  **2. electronic** | ***1. If on paper***  Location of cabinet: Informed consent and ATMP batch dossiers will be kept in the PI's office, room m379.04.149 cabinet 1, shelve 1.  Locking of and access to cabinet is GCP compliant  This cabinet is different from the location of the codelist  ***2.And / If electronic***  [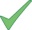](https://www.google.nl/url?q=http://vector.me/search/check-mark&sa=U&ei=33AgU478B6Wd0wXav4GIDA&ved=0CC0Q9QEwBQ&usg=AFQjCNG2vSjGtoxg-z3Ic-vu90eu4Ni7Wg)The data are collected and stored on the secure Castor servers (see for information https://castoredc.com/nl/veiligheid.html)  [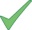](https://www.google.nl/url?q=http://vector.me/search/check-mark&sa=U&ei=33AgU478B6Wd0wXav4GIDA&ved=0CC0Q9QEwBQ&usg=AFQjCNG2vSjGtoxg-z3Ic-vu90eu4Ni7Wg) This folder is password protected  The password is kept safe from unauthorised use  At least two persons have access to this folder, name: van Essen, Peter, van Pinxten-van Orsouw, Elise; and name: Hoogstad-van Evert, Janneke  [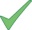](https://www.google.nl/url?q=http://vector.me/search/check-mark&sa=U&ei=33AgU478B6Wd0wXav4GIDA&ved=0CC0Q9QEwBQ&usg=AFQjCNG2vSjGtoxg-z3Ic-vu90eu4Ni7Wg)This folder is different from the location of the codelist | | |
| **Method of coding** | The data are coded by the following method: Each patient is assigned a unique patient study number at enrollment. In trial documents the patient’s identity is coded by patient study number as assigned at enrollment. | | |
| **Location of and access to the codelist**  **1. paper**  **2. electronic** | ***1. If on paper***  Location of cabinet: Trialbureau oncologie, route 460, Kamer 1.14  Locking of and access to cabinet is GCP compliant  ***2. And / If electronic***  The codelist and screeninglog will be signed after inclusion of a patient and saved as PDF file in a folder on the H-server of the Hematology department of the Radboudumc Y:\15 CTI research\Algemeen CTI\klinische studies\screeningslog\INTRO Not in My Documents (folder with z-number)!  *(server instance, localization, folder name including (sub)directory*  This folder is password protected  The password is kept safe from unauthorised use  At least two persons have access to this folder, name: Dolstra, Harry and name: Hoogstad-van Evert, Janneke | | |
| **Data back-ups** | [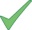](https://www.google.nl/url?q=http://vector.me/search/check-mark&sa=U&ei=33AgU478B6Wd0wXav4GIDA&ved=0CC0Q9QEwBQ&usg=AFQjCNG2vSjGtoxg-z3Ic-vu90eu4Ni7Wg) Yes, frequency: backups are made twice a day on the Castor servers | | |
| **Software used for analysis** | SPSS  SAS  MatLab  Other, specify: | | |
| **Locking* of data and codelist** | Method of locking data: Locking of subjects and blocking user rights in Castor.  Method of locking codelist: The codelist will be signed after inclusion of a patient and saved as PDF file in a folder on the H-server of the Hematology department of the Radboudumc Y:\15 CTI research\Algemeen CTI\klinische studies\screeningslog\INTRO. This folder is protected by a password that is known by Harry Dolstra and Janneke Hoogstad  * freeze data and codes (file/paper) so they become uneditable | | |
| **Archiving of data, codelist and analysis files **** | Location of archiving data: On the H-server of the Hematology department of the Radboudumc Y:\15 CTI research\Algemeen CTI\klinische studies\afgeronde studies (see SOP 'Archivering' of our department) and in the database Castor for 15 years. Thereby a copy of the database will be kept by the datamanagement office on \\Umcfs038\crcndata$\RTC CS Team Integraal\Studies\3. Studies afgerond  Location of archiving codelist: Y:\15 CTI research\Algemeen CTI\klinische studies\screeningslog\INTRO. This folder is protected by a password that is known by Harry Dolstra and Janneke Hoogstad  Location of archiving analysis files: On the sharepoint-site of the Hematology department of the Radboudumc in the folder: https://teamsites.radboudumc.nl/sites/hemat/tb/GYNOC01/Forms After completion of the study a report from Epic will be made and saved as a PDF file on the H driver of hematology onY:\15 CTI research\Algemeen CTI\klinische studies\afgeronde studies .  ** how and where do you securely store all files/paper, e.g. with DANS, network folder, sealed DVD disk in locked cabinet, protected paper archive.... | | |
| **This form is filled in by** | Hoogstad-van Evert, Janneke | | Date: 17-10-18 |

**Optional questions**

The following optional questions on data quality help to:

- get an idea of current practices and ideas in the UMC,
- help to create and improve policy and guidelines with regard to data quality.

You input is highly appreciated! Additional ideas and suggestions can be mailed to [Ariaan.Siezen@radboudumc.nl](mailto:Ariaan.Siezen@radboudumc.nl).

You can skip the questions below if you have already answered them in a previous plan.

| **Quality checks** | I document all procedures and actions during my studies, so as to facilitate future replication by a third party (I have well defined SOPs).  If yes, please explain what kind of SOPs and measures you apply:  If not, explain why:  I check my study data on completeness, validity and consistency, by means of:  manual checks  system checks during data entry  quality reports  other:  If not, explain why:  Please describe your personal definition of data quality:  Would you like more information on this subject? Yes  No  If yes, by means of training/information on CRCN website/other:  Do you think data quality is an important issue? Yes  No  Please explain:  Do you think the Radboud UMC should work at improving data quality?  Yes  No  If yes, what do you think we should do: |
| --- | --- |

**Thank you for taking the time to contribute to better research!**

**Proefpersoneninformatie Natural killer cellen in de buikholte tegen eierstokkanker**

*NL60937.000.17 – versie 1.3 10-09-2018 EudraCTnr 2016-000299-78 pagina 1 van 9*

**Proefpersoneninformatie voor deelname aan medisch-wetenschappelijk onderzoek**

**Natural killer cellen in de buikholte tegen eierstokkanker.**

*Officiële titel: Veiligheid van intraperitoneale toediening van natural killer cellen in de behandeling van recidief ovariumcarcinoom*

**Inleiding**

Geachte mevrouw,

Wij vragen u vriendelijk om mee te doen aan een medisch-wetenschappelijk onderzoek. Meedoen is vrijwillig. Om mee te doen is wel uw schriftelijke toestemming nodig. U ontvangt deze brief omdat u eierstokkanker had en de CA125 waarde in het bloed nu weer stijgt.

Voordat u beslist of u wilt meedoen aan dit onderzoek, krijgt u uitleg over wat het onderzoek inhoudt. Lees deze informatie rustig door en vraag de onderzoeker om uitleg als u vragen heeft. U kunt er ook over praten met uw partner, vrienden of familie.

Algemene informatie over meedoen aan klinisch onderzoek staat in de bijgevoegde brochure ‘Medisch-wetenschappelijk onderzoek’.

Dit onderzoek wordt gedaan door het Radboudumc. Er zullen 12 proefpersonen meedoen. De landelijke toetsingscommissie CCMO heeft dit onderzoek goedgekeurd. Algemene informatie over de toetsing van onderzoek vindt u in de brochure ‘Medisch-wetenschappelijk onderzoek’.

**1. Doel van het onderzoek**

Het doel van dit onderzoek is om uit te zoeken hoe veilig het is om natural killer cellen met en zonder voorbereidende chemotherapie toe te dienen in de buikholte bij vrouwen met teruggekeerde eierstokkanker.

Eierstokkanker is een nare ziekte waarbij bij veel vrouwen de ziekte weer terug keert na de eerste behandeling. Op dit moment krijgen de meeste vrouwen dan opnieuw chemotherapie om een langere overleving te bereiken. Helaas zorgt dit vaak voor veel bijwerkingen en niet voor het volledig verdwijnen van de kanker. Daarom zijn we op zoek naar nieuwe behandelingen.

Natural killercellen zouden zo’n behandeling kunnen zijn. Natural killercellen zijn afweercellen, die heel gericht kankercellen kunnen doden. Deze cellen zijn al veilig en effectief gebleken bij mensen met leukemie. Met dit onderzoek willen we uitzoeken of het ook veilig gegeven kan worden aan vrouwen met eierstokkanker. In tegenstelling tot bij leukemie patiënten zouden we deze cellen het liefst willen geven zonder voorbereidende chemotherapie. Dit laatste is echter nog niet eerder onderzocht en we weten dus niet of dit ook veilig kan, vandaar dat we u nu vragen om mee te doen aan onderzoek waarin we de
